# NLRP3 inflammasome activation contributes to the pathogenesis of cardiocytes aging

**DOI:** 10.18632/aging.203435

**Published:** 2021-08-25

**Authors:** Li-Zhen Liao, Zhi-Chong Chen, Sui-Sui Wang, Wen-Bin Liu, Chang-Lin Zhao, Xiao-Dong Zhuang

**Affiliations:** 1 Guangdong Engineering Research Center for Light and Health, Guangdong Pharmaceutical University, Guangzhou Higher Education Mega Center, Guangzhou, Guangdong, P.R. China; 2 Guangdong Provincial Key Laboratory of Pharmaceutical Bioactive Substances, Guangdong Pharmaceutical University, Guangzhou Higher Education Mega Center, Guangzhou, Guangdong, P.R. China; 3 Cardiovascular Department, The Sixth Affiliated Hospital of Sun Yat-Sen University, Tianhe, Guangzhou, Guangdong, P.R. China; 4 Cardiology Department, The First Affiliated Hospital of Sun Yat-Sen University, Guangzhou, Guangdong, P.R. China

**Keywords:** NLRP3 inflammasome activation, cardiocytes aging, ROS

## Abstract

Objective: The NOD-like receptor protein 3 (NOD-like receptor protein 3, NLRP3) inflammasome is associated with many physiological processes related to aging. We investigated whether NLRP3 inflammasome activation contributes to the pathogenesis of cardiocytes aging dissected the underlying mechanism.

Methods: H9c2 cells were treated with different concentrations of D-galactose (D-gal, 0, 2, 10 and 50 g/L) for 24 hours. The cytochemical staining, flow cytometry and fluorescence microscope analysis were employed to detect the β-galactosidase (β-gal) activity. Western blot analysis was used to detect the age-associated proteins (P53, P21) and NLRP3 inflammasome proteins [NLRP3, apoptosis-associated speck-like protein (ASC)]. Confocal fluorescent images were applied to capture the colocalization of NLRP3 and caspase-1. Intracellular reactive oxygen species (ROS) was measured using 2’7’-dichlorodihydrofluorescein diacetate (DCFH-DA) by flow cytometry and visualized using a fluorescence microscope. The IL-1β, IL-18 and lactate dehydrogenase (LDH) release were also detected.

Results: D-gal induced-H9c2 cells caused cardiocytes’ aging changes (β-gal staining, CellEvent™ Senescence Green staining, P53, P21) in a concentration-dependent manner. NLRP3 inflammasomes were activated, IL-1β, IL-18 and LDH release and ROS generation were increased in the cardiocytes aging progress. When MCC950 inhibited NLRP3 inflammasomes, it attenuated the cardiocytes aging, yet the ROS generation was similar. Inhibition of ROS by NAC attenuated cardiocytes aging and inhibited the NLRP3 inflammasome activation at the same time. NLRP3 inflammasome activation by nigericin-induced cardiocytes cells aging progress.

Conclusions: NLRP3 inflammasome activation contributes to the pathogenesis of cardiocytes aging, and ROS generation may serve as a potential mechanism by which NLRP3 inflammasome is activated.

## INTRODUCTION

Aging is characterized by developing persistent proinflammatory responses that contribute to atherosclerosis, metabolic syndrome, cancer and frailty [1]. It is a significant risk factor for cardiovascular disease. Take heart failure as an example. Approximately 1% of individuals aged over 50 years are affected by heart failure, which doubles with each decade of life [2]. Even in the absence of overt injury, structural and functional changes occur in the heart as it ages, contributing to the increased susceptibility to cardiovascular disease in older adults [3]. The need for interventions to combat age-related cardiac decline is becoming increasingly urgent as the elderly population grows [4]. Although the impact of aging has been extensively studied, little is known regarding the aging processes in cardiocytes. It is critical to understand the pathogenesis and mechanisms of cardiocyte aging to potentially lead to discovering novel therapeutic targets for age-related cardiovascular diseases.

The NOD-like receptor protein 3 (NOD-like receptor protein 3, NLRP3) inflammasome has recently emerged as unexpected stress and metabolic risk marker. It has also been implicated in developing major aging-related diseases such as cardiovascular disease, type 2 diabetes and neurodegenerative [5]. The NLRP3 inflammasome consists of NLRP3, apoptosis-associated speck-like protein (ASC), pro-caspase-1. NLRP3 is an intracellular sensor that detects environmental irritants, microbial motifs and endogenous danger signals [6]. NLRP3 inflammasome assembly leads to releasing the proinflammatory cytokines, IL-1β and IL-18, which are caspase-1-dependent [6]. The NLRP3 inflammasome is associated with many age-dependent diseases [7, 8]. In a lot of aging-associated disorders, the inflammatory response is increased [9, 10]. Yet, it is unclear whether NLRP3 inflammasome influences cardiomyocyte aging. It is suggested that suppression of NLRP3 prevented many age-associated changes in the heart, preserved cardiac function of aged mice and increased lifespan [11]. However, the molecular mechanism by which the NLRP3 inflammasome is regulated during heart aging is largely unknown.

One of the crucial elements for NLRP3 activation is the generation of reactive oxygen species (ROS) [12]. Interestingly, Denham Harman introduced the free radical theory of aging--an excess of ROS damages macromolecules, and the accumulation of such macromolecular damage leads to cellular and organ dysfunction over time [13]. Here, we hypothesize that NLRP3 inflammasome activation contributes to the pathogenesis of cardiocytes aging and ROS generation may serve as a potential mechanism that activates the NLRP3 inflammasome.

## MATERIALS AND METHODS

### Main reagents and antibodies

We obtained the cell culture reagents from Invitrogen (Carlsbad, CA, USA). We purchased D-galactose (D-gal), N-acetyl-L-cysteine (NAC, ROS scavenger) from Sigma-Aldrich (St. Louis, MO, USA), and MCC950 (NLRP3 inflammasome inhibitor), nigericin (NLRP3 inflammasome activator) from MedChem Express (Monmouth Junction, NJ, USA). The senescence β-galactosidase (β-gal) Staining Kit and DCFH-DA probe were purchased from Beyotime (Shanghai, China). The CellEvent™ Senescence Green Flow Cytometry Assay Kit was purchased from Thermo Fisher Scientific (Waltham, MA, USA). Primary antibodies including P53 (1:1000, Cell Signal Technology, Danvers, MA, USA), P21(1:1000, Cell Signal Technology), β-actin(1:1000, Cell Signal Technology), NLRP3 (1:1000, Adipogen, San Diego, CA, USA) and ASC (1:1000, Abcam, UK) were purchased from the above accompanied. The IL-1β, IL-18 and LDH kits were purchased from Solarbio Technology (Beijing, China).

### Cell culture and treatment

H9c2 cells were a ventricular cardiomyocyte cell line. We purchased them from ATCC (Manassas, VA, USA). H9c2 cells were treated with different concentrations of D-gal for 24 hours to mimic cardiocyte aging *in vitro*. H9c2 cells were pre-treated with MCC950 (10 μM) or NAC (1 mM) for 1 hour and then stimulated with D-gal (10 g/L) for 24 h in some experiments. Cells were then processed for cytochemical staining of β-gal, CellEvent™ Senescence Green staining, confocal fluorescent images, flow cytometry and Western blot.

### Cytochemical staining of β-gal activity

To analyze the senescence characterization of H9c2 cells, a senescence β-gal Staining Kit was employed. In brief, H9c2 cells were seeded in a six-well plate. After different treatments for 24 hours, cells were washed with PBS 3 times. The cells were incubated with 1.5 ml fixation buffer for 15 min at room temperature after rinsing with PBS 3 times. We added 1 ml staining mixture to each well. Then we sealed the plate with parafilm and incubated it at 37° C without CO_2_ overnight. The following day, we replaced the staining mixture with 1 ml PBS and observed the blue-stained cells under an optical microscope. We counted the total number of cells and the blue-stained cells for at least 200 cells. At last, the percentage of cells expressing β-gal was calculated.

### Flow cytometry and fluorescence microscope analysis of β-gal

β-galactosidase activity in the H9c2 cells after different treatments was evaluated using the CellEvent™ Senescence Green Flow Cytometry Assay Kit. We followed the manufacture’s recommendations and detected the β-galactosidase activity both by flow cytometry and fluorescence microscope. As for flow cytometry analysis, in brief, we first prepared the working solution. After different treatments, we digested the H9c2 cells with trypsin, washed the cells with 1X PBS, resuspended the cells in 100 μL of fixation solution and incubated for 10 minutes at room temperature. Then we cleaned the cells in 1% BSA in PBS to remove the fixation solution, resuspended the cells in 100 μL of Working Solution, incubated the cells for 1-2 hours at 37° C without CO2, re-suspended the cells in 1% BSA in PBS then analyzed on a flow cytometer using a 488-nm laser and 530-nm/30 filter or similar. The mean fluorescence intensity was measured in each experiment. As for the fluorescence microscope, after different treatments in 6 wells plates, H9c2 cells were fixed with 4% paraformaldehyde for 10 minutes and incubated with 1000 μL of Working Solution for 1-2 hours at 37° C without CO_2_. Then we could observe the senescence-associated-galactosidase expression detected by the CellEvent™ Senescence Green Probe under a fluorescence microscope.

### Western blot

In brief, after being treated with a different stimulus, the protein from H9c2 cells lysates were collected. Western blot procedures were processed as standard protocol with specific antibodies against P53, P21, NLRP3, caspase-1 (1:1000, Abclone, USA)and β-actin (1:1000, CST, Fayetteville, GA, USA) primary antibodies at 4° C overnight and then incubated with secondary antibody (1:15000, CST) for 1h at room temperature. Finally, the membrane was incubated Immobilon ECL Ultra Western HRP Substrate (Millipore, Burlington, MA, USA) and visualized by ChemiDoc™ Touch Imaging System (Bio Rad, Hercules, CA, USA). Western blot image was analyzed and quantified using Image J (NIH, Bethesda, MD, USA). Relative expression of protein was normalized with β-actin expression.

### Confocal fluorescent images

H9c2 cells were seeded in the confocal specific petri dishes. After the different stimuli, the H9c2 cells were fixed in 4% paraformaldehyde and incubated with primary antibodies overnight and with secondary antibodies conjugated to FITC for 2 hours (Santa Cruz Biotechnology, Santa Cruz, CA, USA). The LSM780 confocal fluorescence microscope was used to capture the representative images.

### Intracellular ROS

We detected the intracellular ROS by 2’7’-dichlorodihydrofluorescein diacetate (DCFH-DA) as a fluorescent probe [14]. After the different stimuli, we washed the H9c2 cells with PBS 3 times and incubated them with DCFH-DA (20 μM, Beyotime, China) for 30 min. Then we detected the dichlorodihydrofluorescein (DCF) fluorescence by flow cytometry with an excitation of 485 nm and an emission of 520 nm and captured the typical fluorescence image by a fluorescence microscope.

### IL-1β, IL-18 and lactate dehydrogenase (LDH) release

The release of IL-1β and IL-18 into the supernatant was regarded as an inflammatory response after activation of NLRP3 inflammasomes. H9c2 cardiac cells were cultured in the 96-well plate and grew to the confluence of about 80%. After treatment according to the grouping, we harvested the culture supernatants and detected the IL-1β, IL-18 and LDH levels by the Release Assay Kit according to the manufacturer’s instruction.

### Statistics analysis

All *in vitro* experiments were performed at least three times. Results were expressed as mean ± SD. We analyzed the data by Graphpad Prism 6.02 software. Statistical analysis among multiple groups was carried out by one-way ANOVA followed by a Least Significance Difference (LSD) test using SPSS 17.0 software. p < 0.05 was considered statistically significant.

## RESULTS

### D-gal induced-H9c2 cells caused cardiocytes aging changes in a concentration-dependent manner

**Figure 1:**
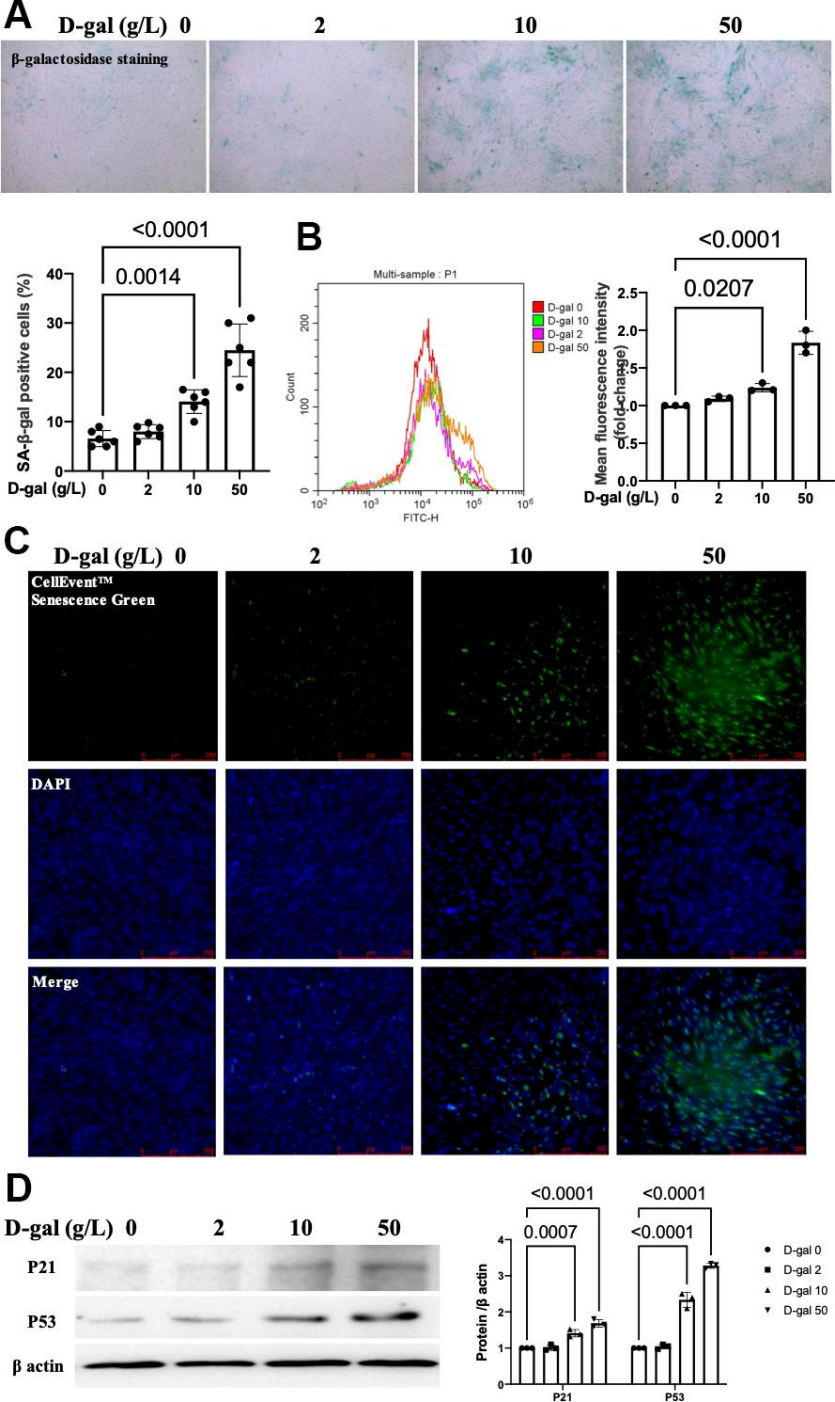
**D-gal induced-H9c2 cells caused cardiocytes aging changes in a concentration-dependent manner.** H9c2 cells were treated with different concentrations of D-gal (0, 2, 10 and 50 g/L) for 24 hours. (**A**) The H9c2 cells senescence induced by D-gal was identified by β-galactosidase staining (100×). Representative bright-field photomicrographs were captured. The blue-stained cells were designated as aging cardiocytes. The blue-stained cells and the total number of cells were counted, and the percentage of cells expressing β-galactosidase was calculated. (**B**) Flow cytometry analysis was applied to detect the β-galactosidase mean fluorescence intensity after different D-gal concentration senescence induction. (**C**) H9c2 cells were stained using the CellEvent™ Senescence Green Probe (200×). An increase in senescence-associated β-galactosidase expression, a hallmark for the onset of senescence, could be detected by the CellEvent™ Senescence Green Probe with a fluorescence microscope. (**D**) The aging-associated proteins (P53, P21) were detected by western blot, and the corresponding quantification was present.

The characteristics of premature aging induced by chronic D-galactose exposure are similar to those in natural aging in rodents [15–17]. Here, we mimicked cardiocytes aging by D-gal induced-H9c2 cells. H9c2 cells were treated with different concentrations of D-gal (0, 2, 10 and 50 g/L) for 24 hours. The β-gal staining (a widely used method to test aging) showed that more β-gal positive cells (blue-stained) were detected in a higher dose D-gal treatment group (Figure 1A). The CellEvent™ Senescence Green staining (Figure 1C) and flow cytometry analysis (Figure 1B) also revealed that D-gal induced-H9c2 cells caused senescence-associated β-gal expression in a concentration-dependent manner. The aging-associated proteins (P53, P21) were increased in a concentration-dependent way. These results indicated that D-gal treatment could mimic cardiomyocyte aging. Based on the above results, we chose 10g/L D-gal treated for 24 hours as the cardiomyocytes aging model in the following experiment.

### NLRP3 inflammasomes were activated in the cardiocytes aging model induced by D-gal

**Figure 2:**
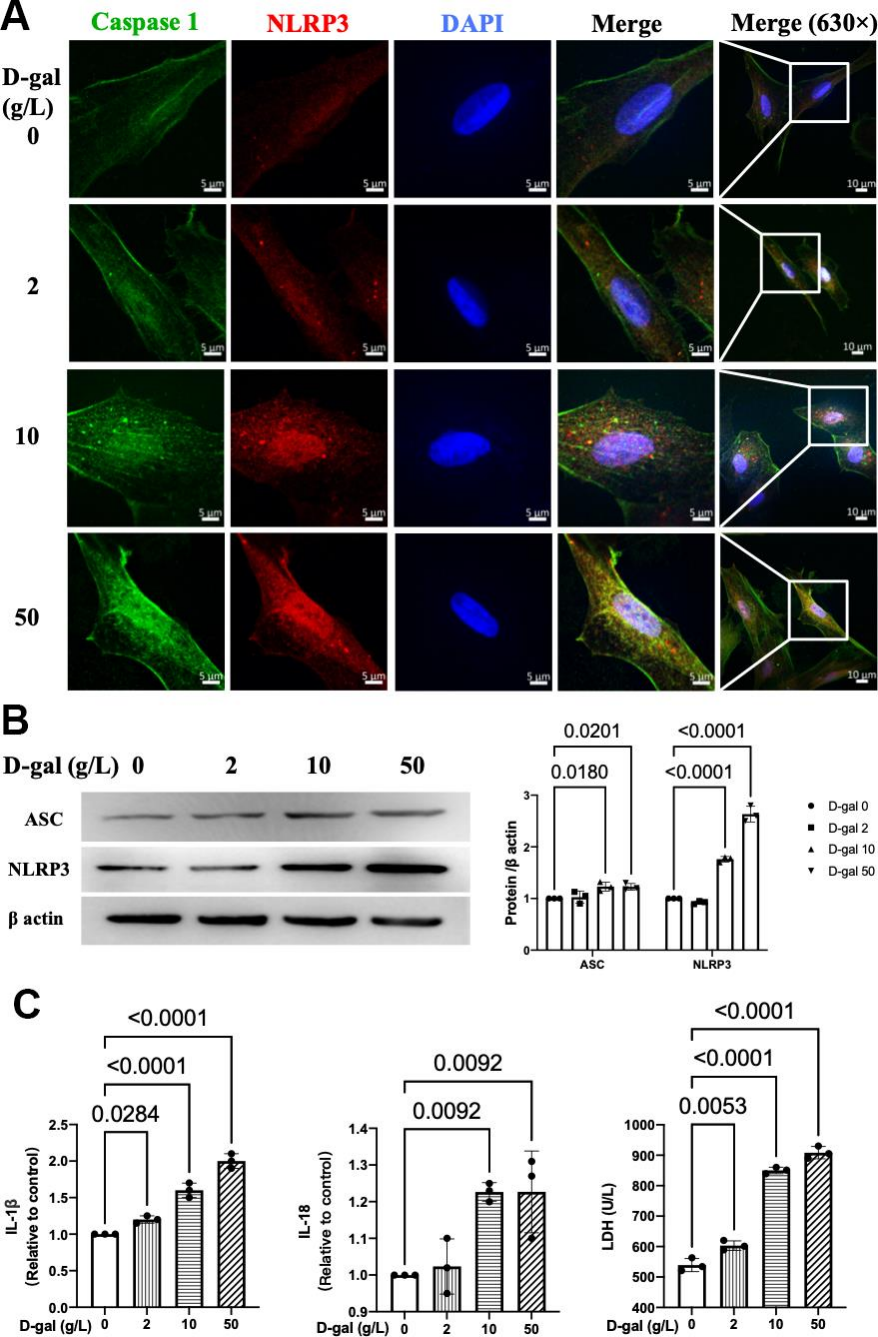
**NLRP3 inflammasomes were activated in the cardiocytes aging model induced by D-gal.** H9c2 cells were treated with different concentrations of D-gal (0, 2, 10 and 50 g/L) for 24 hours. (**A**) Representative confocal fluorescent images showed that D-gal treatment increased the colocalization of NLRP3 (red) and caspase-1 (green) proteins in a concentration-dependent manner. (**B**) Representative immunoblots of the NLRP3 and ASC proteins and the corresponding quantification were shown. (**C**) IL-1β, IL-18 and LDH release levels in cell culture were detected. NLRP3, Nod-like receptor family pyrin domain containing 3; ASC, apoptosis-associated.

There were two main processes when NLRP3 inflammasome was activated. The first one was the transcription of NLRP3 and the precursors of caspase-1 (pro-caspase-1) and IL-1β (pro-IL-1β). The second process was the assembly of NLRP3 with the adaptor protein ASC and pro-caspase-1 [18]. It resulted in the autocleavage of pro-caspase-1 and then the secretion of IL-1β and IL-18 [19]. We further detected whether NLRP3 inflammasomes were activated in the cardiomyocytes aging model. The immunofluorescence studies indicated that the colocalization of NLRP3 (red) and caspase 1 (green) proteins were more significant in a higher D-gal treatment group (Figure 2A). Moreover, the NLRP3 and ASC proteins were also significantly increased accordingly (Figure 2B), indicating that NLRP3 inflammasomes were activated in the cardiocytes aging model induced by D-gal *in vitro*. ELISA measurement was used to determine the IL-1β and IL-18 levels treated with different concentrations of D-gal. Significant increases were observed in a higher D-gal treatment group (Figure 2C). Compared with the control group, the IL-1β level was increased even in the 2 g/L D-gal treatment group. The LDH release level in cell culture was also increased accordingly, indicating D-gal induced cytotoxicity during the cardiocytes aging progress.

### ROS generation was increased in the cardiocytes aging model induced by D-gal

**Figure 3:**
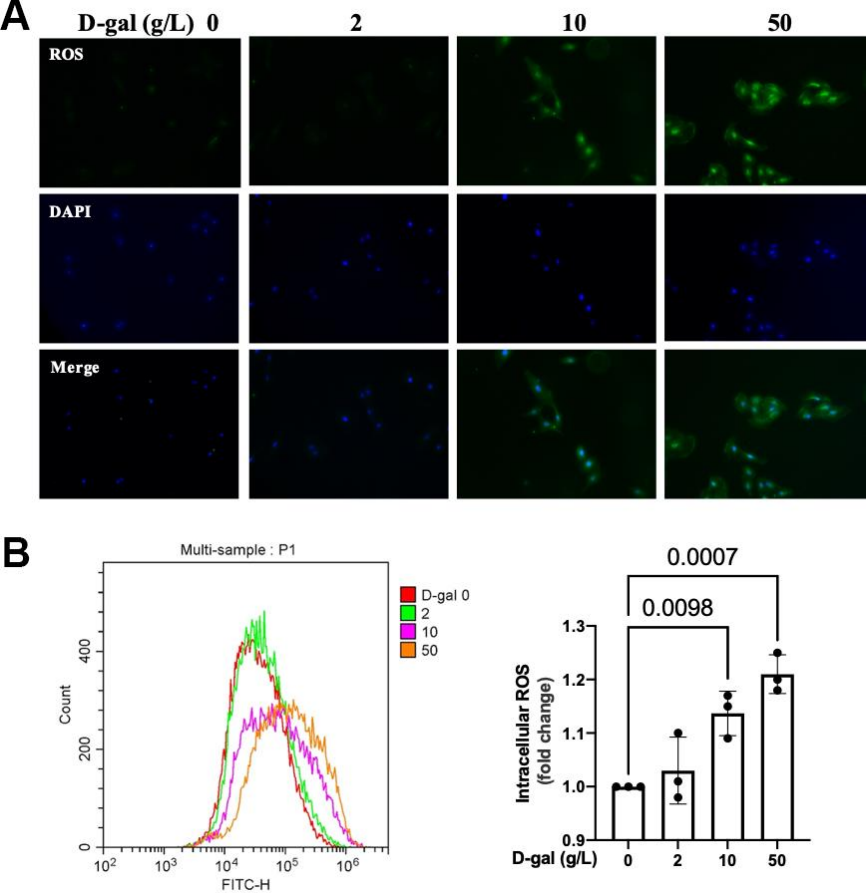
**ROS generation was increased in the cardiocytes aging model induced by D-gal.** H9c2 cells were treated with different concentrations of D-gal (0, 2, 10 and 50 g/L) for 24 hours. (**A**) ROS generation was detected using a DCFH-DA probe. Representative fluorescent images showed that ROS generation was increased in the cardiocytes aging model induced by D-gal *in vitro*. (**B**) Intracellular ROS was quantified by flow cytometry.

The generation of ROS was crucial for NLRP3 activation [12]. We then detected the ROS generation by a DCFH-DA probe. Both the fluorescent images and flow cytometry revealed that the ROS generation was increased in the cardiocytes aging model induced by D-gal (Figure 3).

### Inhibition of NLRP3 by MCC950 attenuated cardiocytes aging induced by D-gal in H9c2 cells

**Figure 4:**
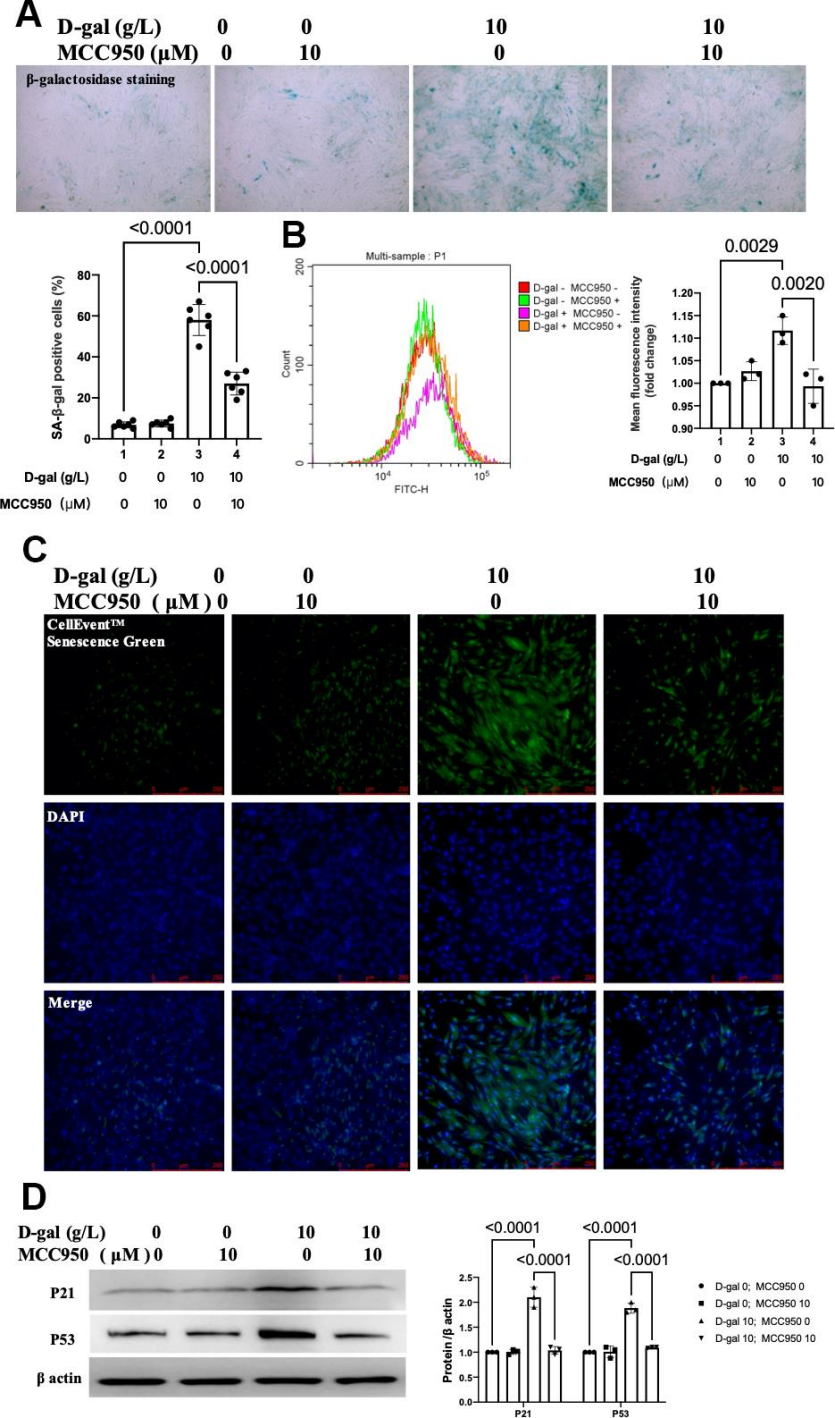
**Inhibition of NLRP3 by MCC950 attenuated cardiocytes aging induced by D-gal in H9c2 cells.** H9c2 cells were pre-treated with or without MCC950 (10μM), a commonly used NLRP3 inhibitor, for 1 hour, and then incubated with or without 10g/L D-gal for 24 hours. (**A**) Representative bright-field photomicrographs showed that MCC950 treatment decreased the percentage of cells expressing β-galactosidase. (**B**) Flow cytometry analysis was applied to detect the β-galactosidase mean fluorescence intensity after the MCC950 treatment. (**C**) The CellEvent™ Senescence Green staining showed that MCC950 treatment decreased the senescence-associated β-galactosidase expression induced by D-gal. (**D**) The aging-associated proteins (P53, P21) were detected by western blot, and the corresponding quantification was present.

MCC950 was one of the most commonly-used NLRP3 inhibitors [20]. To confirm the effect of NLRP3 inflammasomes activation on cardiocytes aging, MCC950 was used to inhibit the NLRP3 activation. The β-gal staining revealed that MCC950 treatment decreased the percentage of blued-stained cells (Figure 4A). Both the CellEvent™ Senescence Green staining and flow cytometry showed that MCC950 treatment decreased the senescence-associated β-gal expression induced by D-gal (Figure 4B, 4C). The aging-associated proteins (P53, P21) were also reduced when MCC950 was added. These results indicated that inhibition of NLRP3 by MCC950 attenuated cardiocytes aging progress.

### MCC950 inhibited NLRP3 inflammasomes in the cardiocytes aging model induced by D-gal

**Figure 5:**
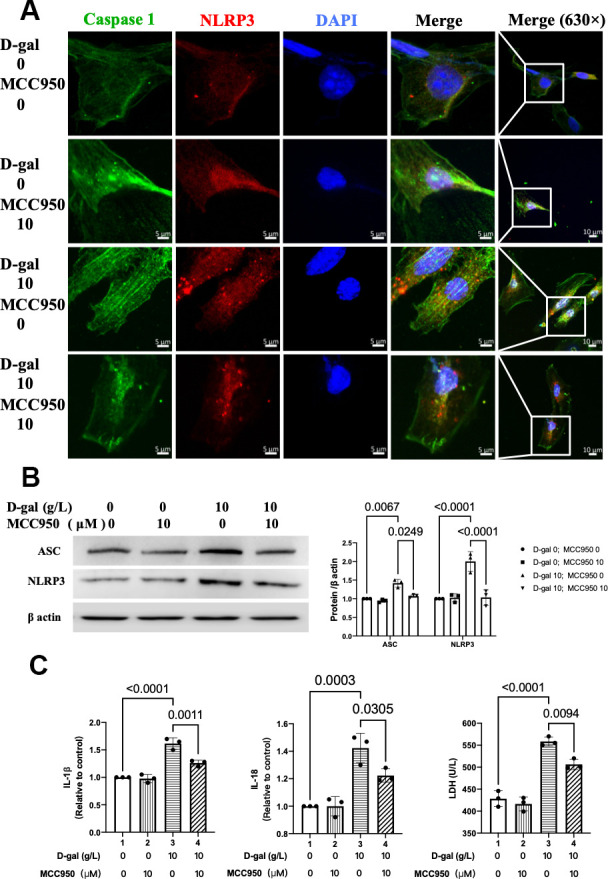
**MCC950 inhibited NLRP3 inflammasomes in the cardiocytes aging model induced by D-gal.** H9c2 cells were pre-treated with or without MCC950 (10μM), a commonly used NLRP3 inhibitor, for 1 hour, and then incubated with or without 10g/L D-gal for 24 hours. (**A**) Representative confocal fluorescent images showed that MCC950 pre-treatment decreased the colocalization of NLRP3 (red) and caspase 1 (green) proteins in the cardiocytes aging model induced by D-gal. (**B**) Representative immunoblots of the NLRP3 and ASC proteins and the corresponding quantification were shown. (**C**) IL-1β, IL-18 and LDH release levels in cell culture were detected. NLRP3, Nod-like receptor family pyrin domain containing 3; ASC, apoptosis-associated speck-like protein.

We then tested the NLRP3 inflammasomes by confocal microscopy. Representative confocal fluorescent images showed that MCC950 pre-treatment decreased the colocalization of NLRP3 (red) and caspase-1 (green) proteins in the cardiocytes aging model (Figure 5A). Besides, the NLRP3 and ASC proteins were also significantly decreased (Figure 5B). Moreover, the IL-1β, IL-18 and LDH release levels in cell culture were also reduced when NLRP3 inflammasomes were inhibited (Figure 5C). These results indicated that NLRP3 inflammasomes and the following inflammatory response were inhibited by MCC950 effectively.

### ROS generation was similar when NLRP3 inflammasomes were inhibited in the cardiocytes aging model induced by D-gal

**Figure 6:**
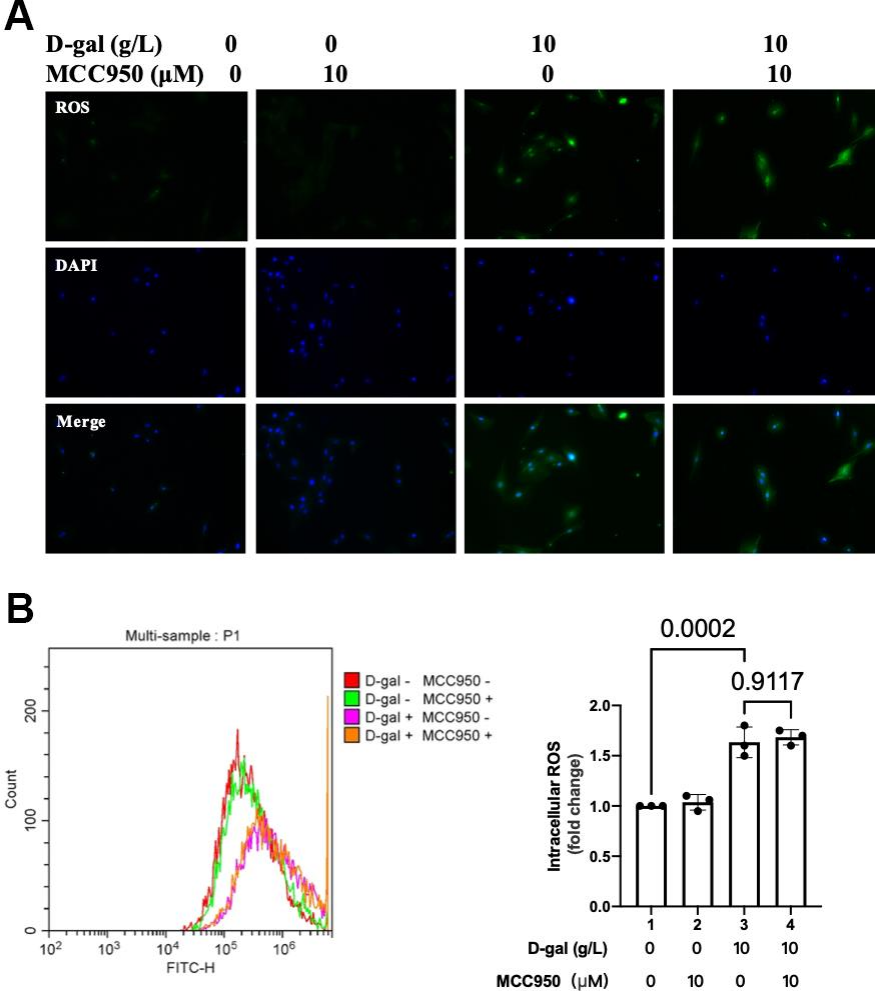
**ROS generation was similar when NLRP3 inflammasomes were inhibited in the cardiocytes aging model.** H9c2 cells were pre-treated with or without MCC950 (10μM), a commonly used NLRP3 inhibitor, for 1 hour, and then incubated with or without 10g/L D-gal for 24 hours. (**A**) ROS generation was detected using a DCFH-DA probe. Representative fluorescent images showed that ROS generation was similar no matter NLRP3 inflammasomes were inhibited or not in the cardiocytes aging model. (**B**) Intracellular ROS was quantified by flow cytometry.

In Figures 2, 3 demonstrated that the NLRP3 inflammasomes were activated, and the ROS generation was increased in the cardiocytes aging progress. We then test when NLRP3 inflammasomes were inhibited, the ROS generation was affected or not. To our surprise, both the fluorescent images and flow cytometry showed that ROS generation was similar no matter NLRP3 inflammasomes were inhibited or not (Figure 6). These results indicated that though both ROS generation and NLRP3 inflammasomes activation contributed to heart aging progress, yet it was likely that the ROS generation came before NLRP3 inflammasomes activation.

### Inhibition of ROS by NAC attenuated cardiocytes aging induced by D-gal in H9c2 cells

**Figure 7:**
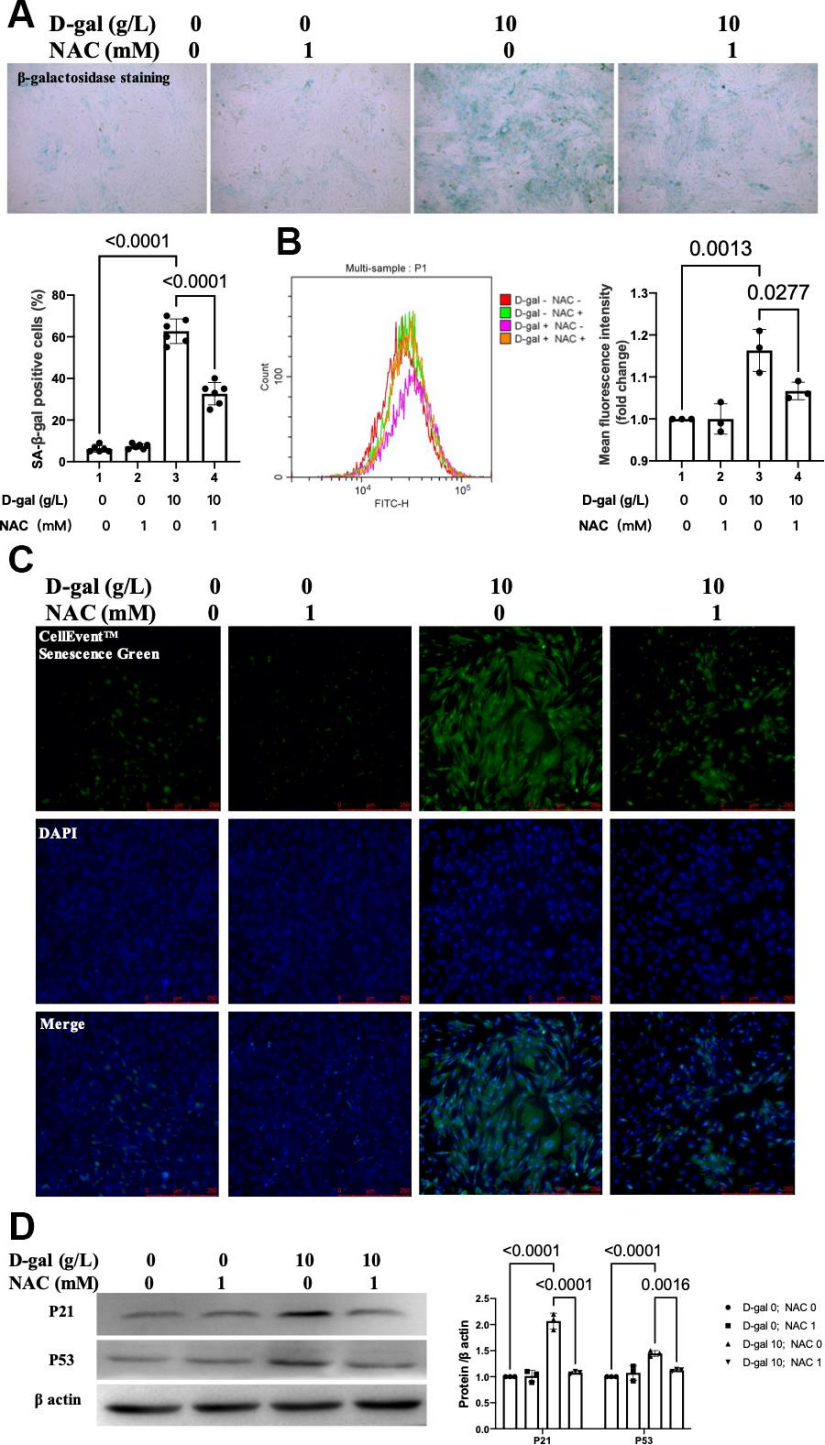
**Inhibition ROS by NAC attenuated cardiocytes aging induced by D-gal in H9c2 cells.** H9c2 cells were pre-treated with or without NAC (1 mM), a commonly used ROS scavenger, for 1 hour and then incubated with or without 10 g/L D-gal for 24 hours. (**A**) Representative bright-field photomicrographs showed that NAC treatment decreased the percentage of cells expressing β-galactosidase. (**B**) Flow cytometry analysis was applied to detect the β-galactosidase mean fluorescence intensity after the NAC treatment. (**C**) The CellEvent™ Senescence Green staining showed that NAC treatment decreased the senescence-associated β-galactosidase expression induced by D-gal. (**D**) The aging-associated proteins (P53, P21) were detected by western blot, and the corresponding quantification was present.

To confirm the effect of ROS generation on cardiocytes aging, NAC was used to scavenge the ROS. The β-gal staining revealed that NAC treatment decreased the percentage of blued-stained cells (Figure 7A). Both the CellEvent™ Senescence Green staining and flow cytometry showed that NAC treatment decreased the senescence-associated β-gal expression induced by D-gal (Figure 7B, 7C). The aging-associated proteins (P53, P21) were also reduced when NAC was added. These results indicated that scavenging of ROS by NAC attenuated the cardiocytes aging progress.

### NAC inhibited NLRP3 inflammasomes in the cardiocytes aging model

**Figure 8:**
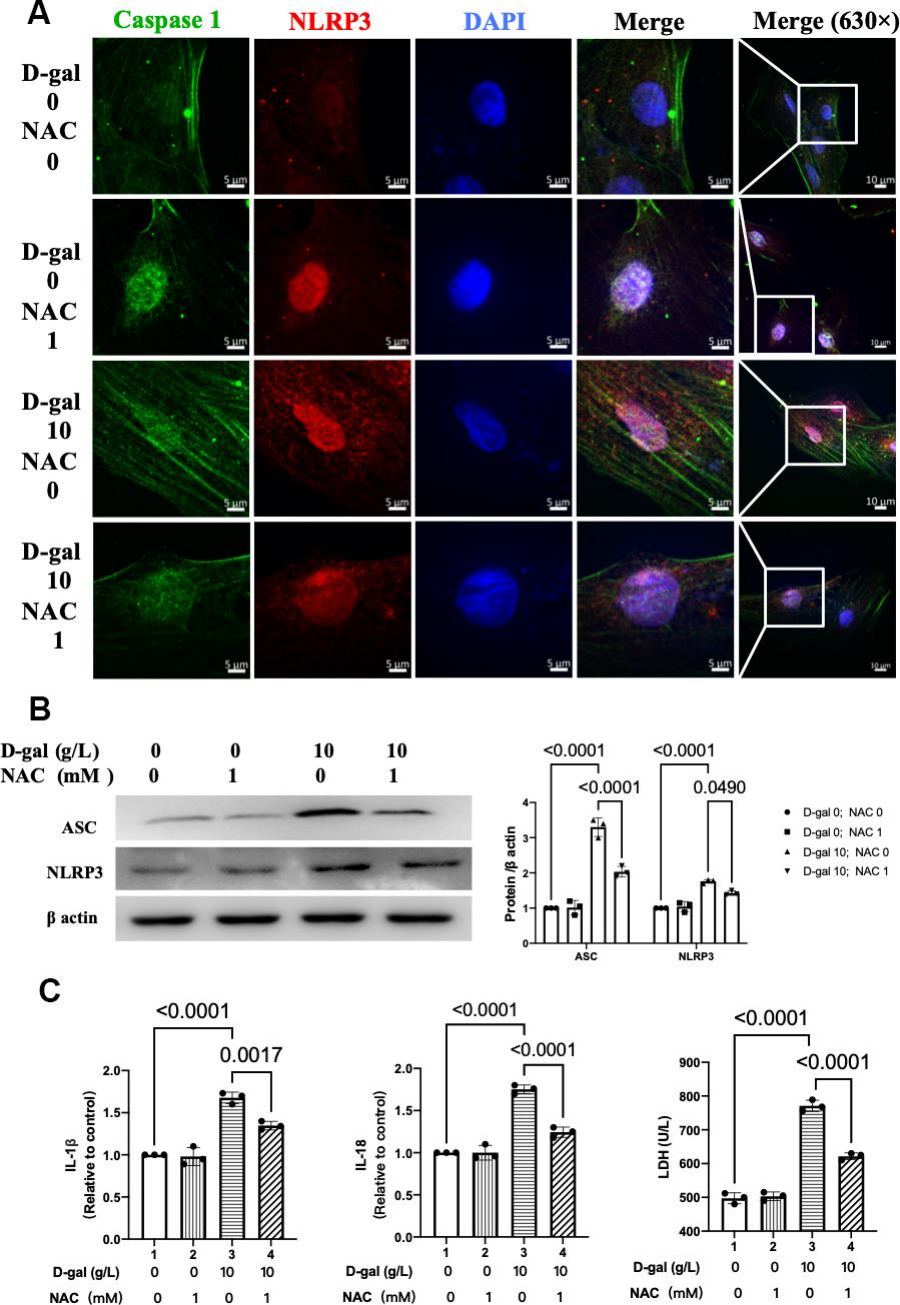
**NAC inhibited NLRP3 inflammasomes in the cardiocytes aging model.** H9c2 cells were pre-treated with or without NAC (1 mM), a commonly used ROS scavenger, for 1 hour and then incubated with or without 10 g/L D-gal for 24 hours. (**A**) Representative confocal fluorescent images showed that NAC pre-treatment decreased the colocalization of NLRP3 (red) and caspase-1 (green) proteins in the cardiocytes aging model induced by D-gal. (**B**) Representative immunoblots of the NLRP3 and ASC proteins and the corresponding quantification were shown. (**C**) IL-1β, IL-18 and LDH release levels in cell culture were detected. NLRP3, Nod-like receptor family pyrin domain containing 3; ASC, apoptosis-associated speck-like protein.

We then further test whether NLRP3 inflammasomes were affected when NAC exerts its cardiocytes aging attenuating effect. Representative confocal fluorescent images showed that NAC pre-treatment decreased the colocalization of NLRP3 (red) and caspase 1 (green) proteins in the cardiocytes aging model induced by D-gal (Figure 8A). Besides, the NLRP3 and ASC proteins were also significantly decreased (Figure 8B). Moreover, the IL-1β, IL-18 and LDH release levels in cell culture were also reduced when NAC was added (Figure 8C). These results indicated that NAC inhibited both NLRP3 inflammasomes and their following inflammatory response.

### NAC decreased ROS generation in the cardiocytes aging model

**Figure 9:**
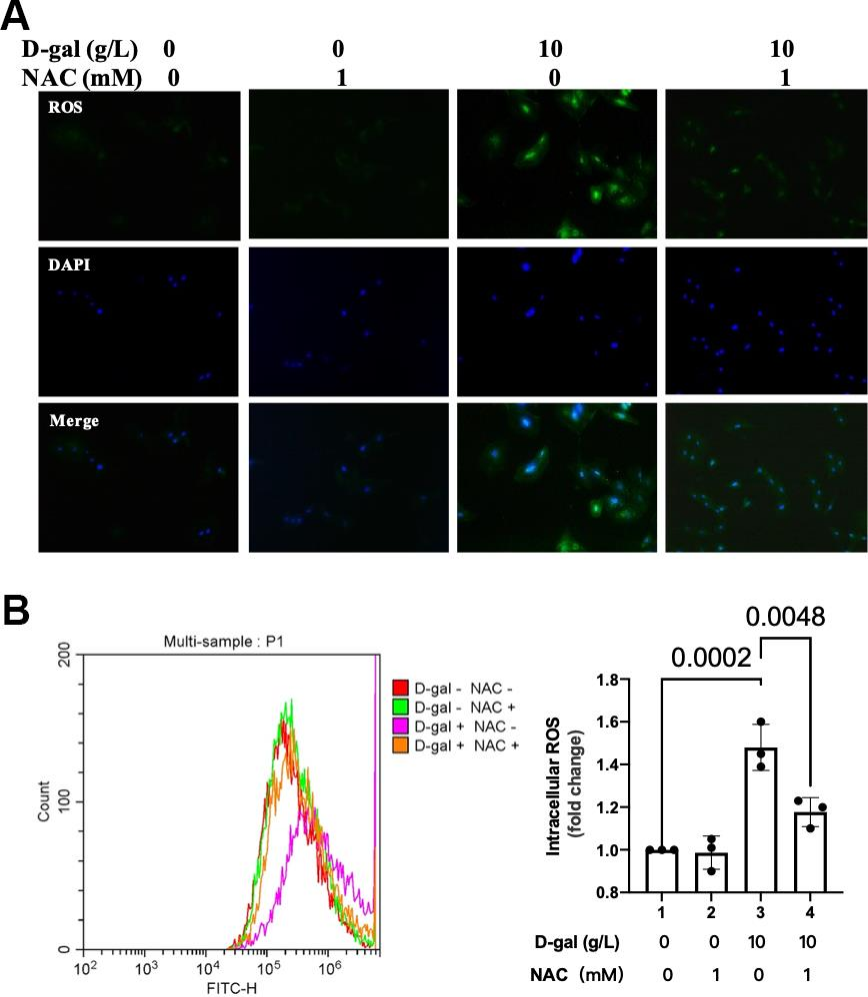
**NAC decreased ROS generation in the cardiocytes aging model.** H9c2 cells were pre-treated with or without NAC (1 mM), a commonly used ROS scavenger, for 1 hour, and then incubated with or without 10 g/L D-gal for 24 hours. (**A**) ROS generation was detected using a DCFH-DA probe. Representative fluorescent images showed that NAC decreased ROS generation in the cardiocytes aging model induced by D-gal *in vitro*. (**B**) Intracellular ROS was quantified by flow cytometry.

In the next step, we tested the ROS generation when NAC was added during the cardiocytes aging progress. Both the fluorescent images and flow cytometry showed that NAC significantly decreased ROS generation in the cardiocytes aging model (Figure 9).

### NLRP3 inflammasome activation by nigericin induced cardiocytes cells aging

Figure 2 demonstrated that the NLRP3 inflammasomes were activated in the cardiocytes aging model induced by D-gal *in vitro*. Nigericin was reported as a commonly used NLRP3 activator [20]. We were curious to figure out when NLRP3 inflammasome was activated by nigericin and whether it affected the aging progress of the cardiocytes. The β-gal staining revealed that nigericin treatment increased the percentage of blue-stained cells (Supplementary Figure 1A). Both the CellEvent™ Senescence Green staining and flow cytometry showed that nigericin treatment increased the senescence-associated β-galactosidase expression (Supplementary Figure 1B, 1C). Besides, the aging-associated proteins (P53, P21) were also raised accordingly (Supplementary Figure 1D). These results indicated that NLRP3 inflammasome activation contributed to heart aging progress.

### NLRP3 inflammasomes were activated by nigericin in cardiocytes

We then tested the NLRP3 inflammasomes by confocal microscopy. Representative confocal fluorescent images showed that nigericin pre-treatment increased the colocalization of NLRP3 (red) and caspase 1 (green) proteins in the cardiocytes aging model induced by D-gal (Supplementary Figure 2A). Besides, the NLRP3 and ASC proteins were also significantly increased (Supplementary Figure 2B). Moreover, the IL-1β, IL-18 and LDH release levels in cell culture were also raised when NLRP3 inflammasomes were activated (Supplementary Figure 2C). These results indicated that NLRP3 inflammasomes were and their following inflammatory responses were triggered by nigericin effectively.

## DISCUSSION

Many molecular changes both inside and outside the cardiomyocyte were involved in the cardiac aging process [21]. Here, we demonstrated for the first time that NLRP3 inflammasome activation contributes to the pathogenesis of cardiocytes aging, and ROS generation may serve as a potential mechanism by which NLRP3 inflammasome is activated based upon the following 3 facts. First, NLRP3 inflammasomes were activated, and ROS generation was increased in the cardiocytes aging model induced by D-gal. Second, inhibition of NLRP3 by MCC950 attenuated cardiocytes aging but did not affect the ROS generation. Last, inhibition of ROS by NAC attenuated cardiocytes aging and inhibited the NLRP3 inflammasomes activating simultaneously.

As the elderly segment of the world population increases, it is critical to understand the changes in cardiac structure and function during the normal aging process [22]. Cardiac aging is characterized by structural and functional changes that are caused by alterations in fundamental cardiomyocyte functions [23]. Cellular senescence is a permanent state of cell cycle arrest that promotes tissue remodeling during development and after injury [14]. Yet little is known during the aging processes in cardiocytes. It is crucial to understand the mechanisms of cardiocyte aging to develop novel treatments for cardiac pathology. D-gal is one of the substances used to instigate aging in various models, and techniques involving this have been widely used since 1991 [15]. In our study, H9c2 cells were treated with different concentrations of D-gal (0, 2, 10 and 50 g/L) for 24 hours to mimic the cardiocytes aging progress. As expected, the β-gal staining, the CellEvent™ Senescence Green staining, flow cytometry analysis and the aging-associated proteins (P53, P21) results (Figure 1) indicated that were D-gal treatment could mimic cardiomyocytes aging progress. In another study *in vitro*, it was reported that 10 g/l of D-galactose was added to the H9c2 cells for 48 hours to establish an aging model [24]. The cardiocytes aging changes happened 1 day later than us, even with the same D-gal concentration (10 g/L). We analyzed that it might due to different senescence-associated hallmarks were detected. We tested the β-gal expression by both β-gal staining and CellEvent™ Senescence Green staining and noticed aging-associated proteins (P53, P21). Meanwhile, Chen J et al. mainly focused on the cell viability apoptosis and autophagy changes induced by D-gal [24]. Because we evaluated cardiocytes aging with different indicators, different D-gal induced-time was acceptable. We chose 10g/L D-gal treated for 24 hours as the cardiomyocytes aging model in the following experiment.

NLRP3 inflammasome, as a participant of the inflammatory immune response, is closely related to cardiovascular diseases [25]. Numerous studies have confirmed that NLRP3 inflammasome is involved in the occurrence and development of myocardial I/R injury, cardiomyopathy, arrhythmia, and other diseases [7, 26–28].

As for cardiac aging *in vivo*, the harmful effects would attenuate if NLRP3 was inhibited [11]. Moreover, the absence of NLRP3 (NLRP3-knockout mice) prevented age-related mitochondrial dynamic alterations in cardiac muscle with minimal effects in cardiac autophagy during aging [8]. The above evidence indicated that activation of NLRP3 inflammasome might exacerbate cardiac aging. Our results also found that NLRP3 inflammasomes were activated in the cardiocytes aging model induced by D-gal (Figure 2). In the next step, we further inhibited NLRP3 with MCC950 (Figure 5). It did attenuate the cardiocyte’s aging (Figure 4). To confirm the role of NLRP3 inflammasome activation in cardiocytes cells aging on the other aspect, we further activated NLRP3 inflammasome with nigericin. As we expected, NLRP3 inflammasome activation (Supplementary Figure 2) did induce cardiocytes cells aging progress (Supplementary Figure 1). Our results provided evidence that NLRP3 inflammasome activation contributes to the pathogenesis of cardiocytes aging, and in cardiac aging, NLRP3 inhibition attenuated the associated decreased function.

The NLRP3 inflammasome activation will trigger the cleavage of pro-interleukin (IL)-1β and pro-IL-18, finally promoting the inflammatory process [29]. Diverse stimuli can activate the NLRP3 inflammasome, such as reactive oxygen species (ROS), mitochondrial dysfunction, and ionic flux [30]. The danger signals for activating NLRP3 inflammasome are extensive, primarily ROS, which act as an intermediate trigger to activate NLRP3 inflammasome, exacerbating subsequent inflammatory cascades and cell damage [29]. Specific ROS levels have been demonstrated as potentially critical for induction and maintenance of the cell senescence process. A causal connection between ROS, aging, age-related pathologies, and cell senescence is studied intensely [31, 32].

Oxidative stress due to excessive ROS has also been shown to play an essential role in the pathophysiology of cardiac aging. Compared with adult mice (12 months), abnormal higher levels of oxidative stress in aging mice (24 months) were found [33]. Our study demonstrated that ROS generation was increased in the cardiocytes aging model (Figure 3). An interesting finding of our research is that when we inhibited ROS by NAC (Figure 9), the cardiocytes aging progress was attenuated (Figure 7), and NLRP3 inflammasomes activation induced by D-gal was also inhibited (Figure 8) at the same time. These results indicated that ROS generation might serve as a potential mechanism by which NLRP3 inflammasome is activated.

As far as we know, we are the first demonstrating that NLRP3 inflammasome activation contributes to the pathogenesis of cardiocytes aging, and ROS generation may serve as a potential mechanism by which NLRP3 inflammasome is activated in cardiocytes aging progress. It provided a new therapeutic strategy for preventing and alleviating cardiocyte aging.

## Supplementary Material

Supplementary Figures
