# Supplementary material for: NLRP3 inflammasome activation contributes to the pathogenesis of cardiocytes aging
Source: Aging (Albany NY). 2021 Aug 25;13(16):20534–51. doi: 10.18632/aging.203435 (PMC8436929; doi:10.18632/aging.203435)
Supplement: Supplementary Figures [file aging-13-203435-s001.pdf]

SUPPLEMENTARY FIGURES

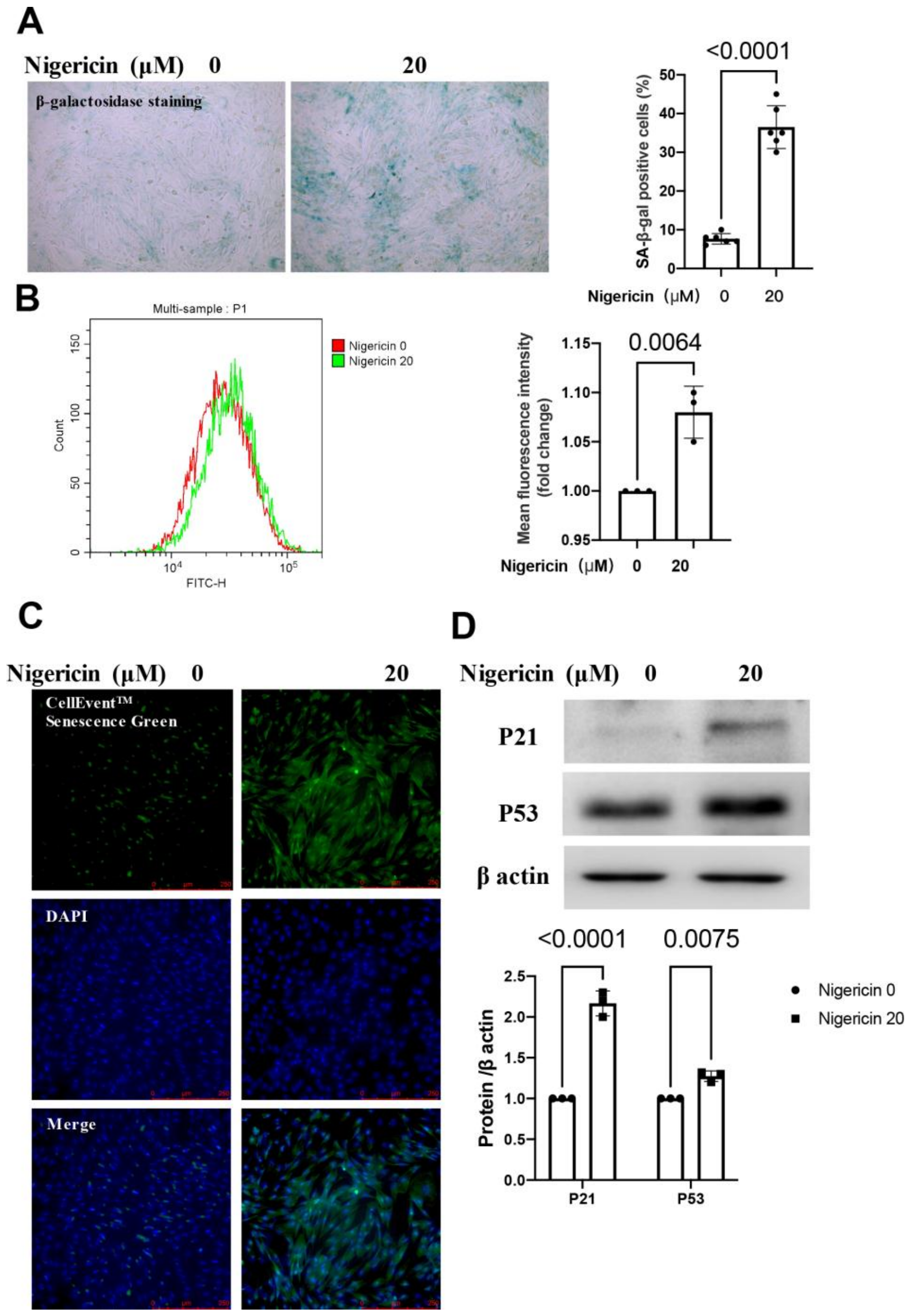

Supplementary Figure 1. NLRP3 inflammasome activation by nigericin induced cardiocytes aging. H9c2 cells were pre-treated with or without nigericin (20μM), a commonly used NLRP3 activator, for 1 hour, and then incubated with standard medium for 24 hours. (**A**) Representative bright-field photomicrographs showed that nigericin treatment increased the percentage of cells expressing β-galactosidase. (**B**) Flow cytometry analysis was applied to detect the β-galactosidase mean fluorescence intensity after the nigericin treatment. (**C**) The CellEvent™ Senescence Green staining showed that nigericin treatment increased the senescence-associated β-galactosidase expression. (**D**) The aging-associated proteins (P53, P21) were detected by western blot, and the corresponding quantification was present.

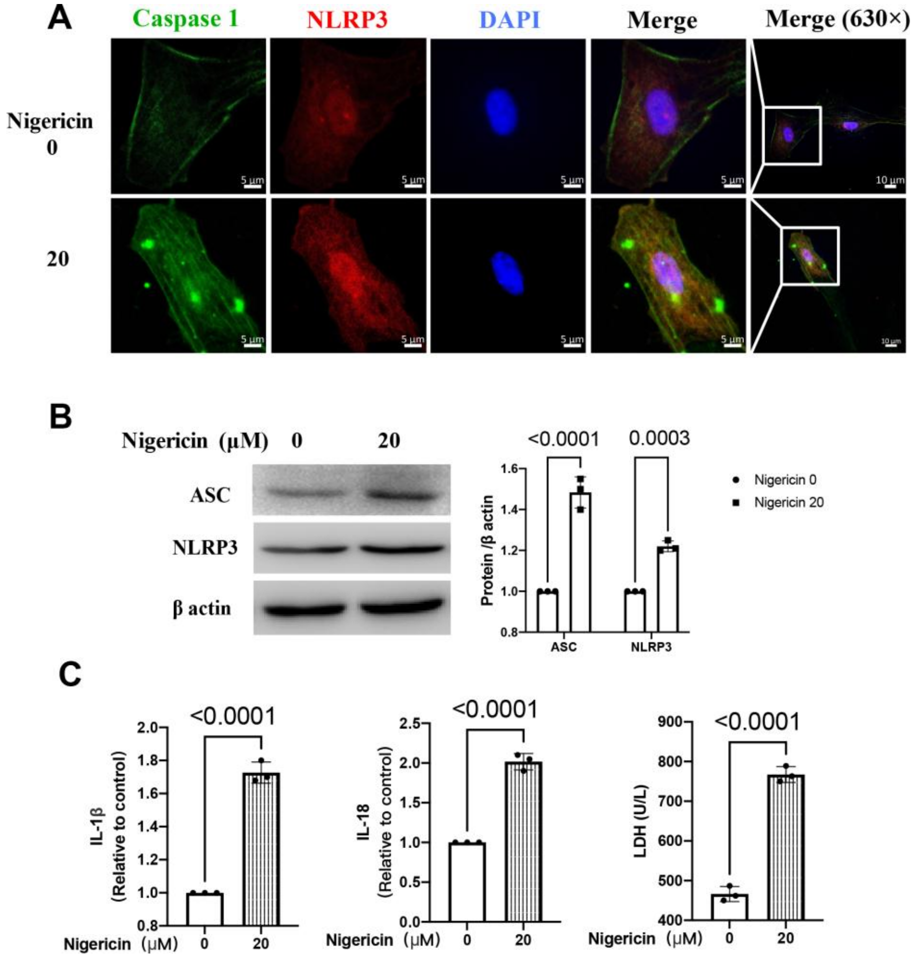

Supplementary Figure 2. NLRP3 inflammasomes were activated by nigericin in cardiocytes. H9c2 cells were pre-treated with or without nigericin (20μM), a commonly used NLRP3 activator, for 1 hour, and then incubated with standard medium for 24 hours. (**A**) Representative confocal fluorescent images showed that nigericin treatment increased the colocalization of NLRP3 (red) and caspase-1 (green) proteins in a concentration-dependent manner. (**B**) Representative immunoblots of the NLRP3 and ASC proteins and the corresponding quantification were shown. (**C**) IL-1β, IL-18 and LDH release levels in cell culture were detected. NLRP3, Nod-like receptor family pyrin domain containing 3; ASC, apoptosis-associated speck-like protein.
